# 3D virtual histology at the host/parasite interface: visualisation of the master manipulator, *Dicrocoelium dendriticum*, in the brain of its ant host

**DOI:** 10.1038/s41598-018-26977-2

**Published:** 2018-06-05

**Authors:** Daniel Martín-Vega, Amin Garbout, Farah Ahmed, Martina Wicklein, Cameron P. Goater, Douglas D. Colwell, Martin J. R. Hall

**Affiliations:** 1 Department of Life Sciences, Natural History Museum, SW75BD London, UK; 2 Department of Life Sciences, University of Alcalá, 28805 Alcalá de Henares, Madrid Spain; 3 Imaging and Analysis Centre, Natural History Museum, SW75BD London, UK; 4 Department of Neuroscience, Physiology and Pharmacology, University College London, WC1E 6BT London, UK; 5 Department of Biological Sciences, University of Lethbridge, Lethbridge, Alberta, TIK 3M4 Canada; 6 Agriculture and Agri-Food Canada, T1J 4B1 Lethbridge, Alberta, Canada

## Abstract

Some parasites are able to manipulate the behaviour of their hosts to their own advantage. One of the most well-established textbook examples of host manipulation is that of the trematode *Dicrocoelium dendriticum* on ants, its second intermediate host. Infected ants harbour encysted metacercariae in the gaster and a non-encysted metacercaria in the suboesophageal ganglion (SOG); however, the mechanisms that *D. dendriticum* uses to manipulate the ant behaviour remain unknown, partly because of a lack of a proper and direct visualisation of the physical interface between the parasite and the ant brain tissue. Here we provide new insights into the potential mechanisms that this iconic manipulator uses to alter its host’s behaviour by characterising the interface between *D. dendriticum* and the ant tissues with the use of non-invasive micro-CT scanning. For the first time, we show that there is a physical contact between the parasite and the ant brain tissue at the anteriormost part of the SOG, including in a case of multiple brain infection where only the parasite lodged in the most anterior part of the SOG was in contact with the ant brain tissue. We demonstrate the potential of micro-CT to further understand other parasite/host systems in parasitological research.

## Introduction

Animals infected with parasites often behave differently relative to those that are not. In some cases, infected hosts act spectacularly differently relative to their uninfected counterparts^1–3^. Terrestrial crickets infected with hairworms seek and then plunge into water. Some roundworms chauffeur their ground-hugging ant hosts into the tree canopy where they wave their conspicuous red abdomens to mimic edible berries. Fungi can infect ants and enslave their hosts to die in the precise locations on a plant that make them most likely to transmit spores. It is these bizarre behavioural alterations that have captivated biologists for many years. Yet the mechanisms that underlie these changes in host behaviour are almost completely unknown. Recent reviews consider this knowledge gap to be the key impediment to our understanding of this pervasive natural phenomenon^3^ and the key roadblock in our ability to distinguish altered host behaviours that arise due to mere ‘sickness’, from host alterations that enhance parasite reproduction and development.

The absurd behaviours of ants infected with larvae of the trematode, *Dicrocoelium dendriticum* (Rudolphi), were described by German parasitologists in the 1970’s^4,5^. Similar behaviours occur at sites in North America where the parasite has been introduced^6,7^. In a region of emergence in Cypress Hills Park in Alberta, Canada, infected ants (*Formica aserva* Forel) leave their nests during cool evenings to affix their mandibles onto the petals of flowers. This attachment to vegetation presumably facilitates the transmission of larval flukes into elk, deer, and beef cattle in this region^8^. The ants remain attached to flower petals overnight, but detach the next day if air temperature exceeds 18–20 °C. Ants remain attached for up to 7 days if temperatures stay cool. During attachment, ants do not feed or defend themselves from predators. Marked ants have been observed returning to their nest following detachment, where they demonstrate typical worker activities, then attach to the very same petal the next day (CPG/DDC, unpublished observations).

**Figure 1:**
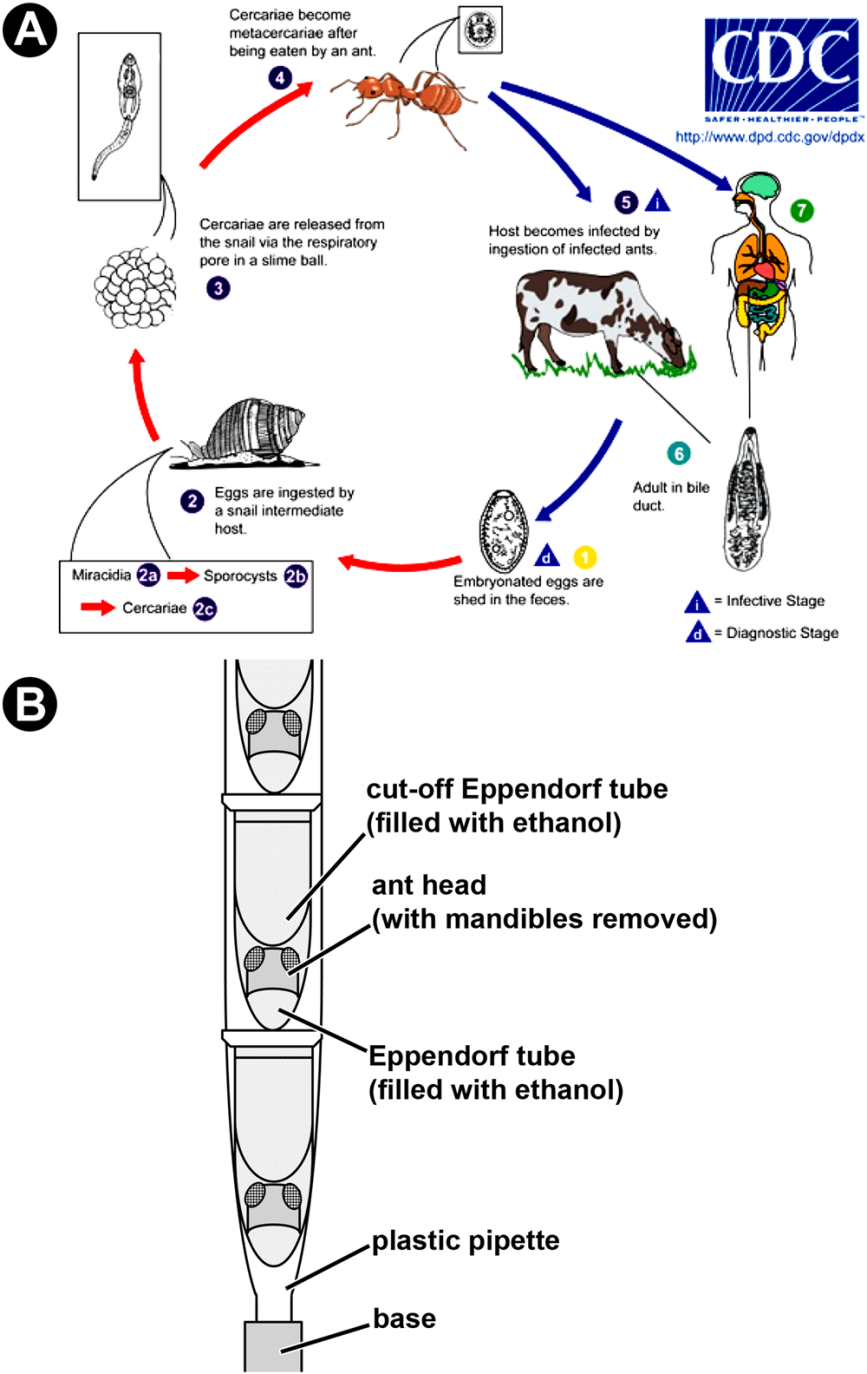
(**A**) Life cycle of *Dicrocoelium dendriticum*, image courtesy of the CDC (Centers for Disease Control and Prevention) at https://www.cdc.gov/dpdx/dicrocoeliasis/index.html. See Introduction for details. (**B**) Schematic illustration of the set-up used to scan specimens.

The life cycle of *D. dendriticum* (Fig. 1A) might indeed look like a complex series of unlikely events. Following ingestion by a terrestrial snail, *D. dendriticum* eggs hatch and miracidia move to the snail digestive gland, where they transform into asexually reproductive mother sporocysts, which produce daughter sporocysts, which produce in turn numerous cercariae. Mature cercariae move to the snail respiratory chamber, where they are released into the environment within snail-produced, ant-appetising slime balls. When an ant ingests one of these slime balls, cercariae pierce the crop wall and one of them, or rarely two, invades and occupies the suboesophageal ganglion (SOG)^4,5^. The rest of the cercariae encyst in the gaster, becoming quiescent infective metacercariae^9^. The non-encysted metacercaria lodge in the brain where the so-called ‘Hirnwurm’ or ‘brainworm’ manipulates the ant behaviour.

Determining the mechanisms underlying the ‘attach-detach-repeat’ manipulation in *Dicrocoelium*-infected ants is a challenging task. One possibility is that a parasite that occupies a large proportion of an ant’s SOG could influence a range of host behaviours by its physical association with particular ganglia. Similar arguments have been made for other parasites that reside within the central nervous system (CNS) of their hosts such as *Toxoplasma* in rodents^10^, rabies virus in carnivores^11^, and metacercariae in the brains of fish^12^. But site-selection within the SOG cannot explain the attachment/detachment dichotomy, nor can it explain the long periods of apparently normal behaviour that occur when temperatures exceed 20 °C. Thus, if mechanically-induced dysfunction of the SOG is important, it must involve a temporary, temperature-dependent change in the morphology or orientation of the larva, resulting in a change in its physical contact with the ant brain tissues. Alternatively, or additionally, there could be temporary neurochemical interference with key components of the CNS. Studies designed to test the mechanical versus neurochemical hypotheses for the manipulation of *D. dendriticum*-infected ants would provide important, albeit indirect, advances regarding underlying mechanisms. Direct observations of the *D. dendriticum*/SOG interface would provide additional and fundamental insight into the nature of this radical behavioural manipulation.

Modern imaging tools have the potential to revitalise and revolutionise our understanding of key processes occurring at the host-parasite interface^13^. Micro-computed tomography (micro-CT), for example, provides a non-invasive and less time-consuming tool to traditional histology that has been used in a wide range of biological and medical disciplines^14–16^. Here, a sample is rotated 360° thereby enabling X-ray images to be taken from different angles, so that virtual sections can be calculated from the resulting X-ray projections^15,16^. Lee *et al*.^17^ used CT and micro-CT virtual serial sections to visualise the host-parasite interface in the bronchi of dogs infected with adults of the lung fluke *Paragonimus westermani* Kerbert. Noever *et al*.^18^ used micro-CT to visualise the *in situ* root-like network of a rhizocephalan barnacle inside the integument of its crustacean host. In general, micro-CT methodology is becoming progressively more accessible to researchers combined with greatly improved resolution through the development of scanning systems capable of the submicron voxel size level^15^.

The objective of the present study was to use non-invasive virtual micro-CT and three-dimensional visualisation to characterize the *Dicrocoelium*/SOG interface in infected *Formica aserva*. For comparison, we used the same micro-CT methodology to visualise *D. dendriticum* metacercariae that are located in the body cavity. Our aim was to demonstrate the potential of micro-CT to visualise the host/parasite interface *in situ* as a tool to provide insight into the potential mechanisms that this iconic manipulator uses to so radically alter its host’s behaviour.

## Methods

### Sample collection and preservation

Samples of *Formica aserva* Forel (Hymenoptera: Formicidae) were collected on 8 July, 2016 and 24 August, 2016 between 10:00 a.m. and 12:00 p.m. from a known site of *D. dendriticum* infection in Cypress Hills Interprovincial Park, Alberta^7^. This site contained at least 4 *F. aserva* nests, each with both uninfected and infected workers. Infection characteristics within the 4 nests have been monitored annually since 2010^6,7^. A sample of 20 workers found attached to flowers adjacent to 2 nests was collected on each date, together with a sample of 20 uninfected ants collected directly from the nests. Samples were placed in individual plastic vials, fixed in 70% ethanol, and shipped to the Natural History Museum (London, UK), where they were stored at 4 °C until they were processed for staining and mounting.

### Sample preparation and scanning

A total of 18 ant heads was randomly selected for micro-CT scanning, 12 infected and 6 uninfected. The selected ants were decapitated and the mandibles were removed with a scalpel under a stereomicroscope to enhance the penetration of the staining solution into the brain^14^. Ant heads were then stained by immersion in 1% phosphotungstic acid (PTA) solution (1 mg/ml concentration in 70% ethanol) in individual plastic vials for 7 days. During the staining period, the plastic vials were kept at 4 °C. In addition, the gasters of 4 infected and 2 uninfected decapitated ants were also removed from the ants by scalpel and stained by immersion in 1% PTA solution for 7 days under the same storage conditions. Lastly, the legs of a single infected ant were removed and the full ant body was stained by immersion in 1% PTA solution for 14 days under the same storage conditions. All the scanned ant specimens were approximately of equal size (*c*. 6–7 mm in length).

In preparation for micro-CT scanning, the samples were placed in individual Eppendorf tubes containing 70% ethanol. The conical bottom part of another Eppendorf tube was cut off and placed inside the tube over the sample, thereby gently restraining it against the bottom of the tube in order to minimise the sample moving within the tube during the rotation produced by scanning. Three Eppendorf tubes, each containing one sample, were stacked inside a plastic pipette (Fig. 1B) and scanned in a Zeiss Versa 520 system with 4x optical magnification, using a Zeiss proprietary LE6 filter and exposure set to 6 secs. Current (40–80 μA) and voltage (50–75 kV) were adapted to each individual sample to optimise contrast and signal/noise ratio, and the resulting projections were reconstructed with a voxel (=volume element) size of 1.7–1.9 μm^3^. Each voxel, the smallest volume reconstructed, has a value giving the opacity of its contents to X-rays (radio density). A multi-scale imaging approach was taken for selected samples, so two infected ant heads (one with three non-encysted metacercariae and one with a single non-encysted metacercaria) were rescanned with 20x optical magnification and exposure set to 15 secs, zooming in on the SOG. In addition, an infected ant head with three encysted metacercariae was also rescanned with 40x optical magnification and exposure set to 25 secs, zooming in one of the cysts. For these ‘zoom’ scans, a Zeiss proprietary LE4 filter was used, whereas current (50–80 μA) and voltage (81–88 kV) were again adapted to each individual sample to optimise contrast and signal/noise ratio. Sample movement during rotation in ‘zoom’ scans impeded accurate reconstruction at that resolution. To minimise this problem, three samples were transferred to Eppendorf tubes containing viscous, standard alcohol-based hand sanitiser and rescanned once again. The resulting projections were reconstructed with a voxel size of 0.4–0.8 μm^3^. For all the scans, image acquisition and volumetric reconstruction were performed using Zeiss software, Scout-and-Scan Control System and Reconstructor. The datasets generated during the current study are available from the corresponding author on reasonable request.

Reconstructed volumetric data were imported into VG Studio Max 2.2 (Volume Graphics Gmbh, Heidelberg, Germany), where slice stacks were rendered, reoriented, and visualised in the three principal planes of sectioning (cross, horizontal and sagittal). The reconstructed data were also loaded into Avizo 9.2. (Visualization Sciences Group, Bordeaux, France) for data segmentation, 3D visualisation and volumetric measurements. Segmentation of selected parasites was performed manually, using the ‘Limited range only’ option to specify a range of grey values. Volumetric measurements of segmented materials were performed using the ‘Material Statistics’ module. For ant brain terminology, although Ito *et al*.^19^ recommended the more accurate term ‘gnathal ganglion’ to replace the term ‘suboesophageal ganglion’ (SOG), we used the latter for consistency with previous literature on *D. dendriticum*.

## Results

**Figure 2:**
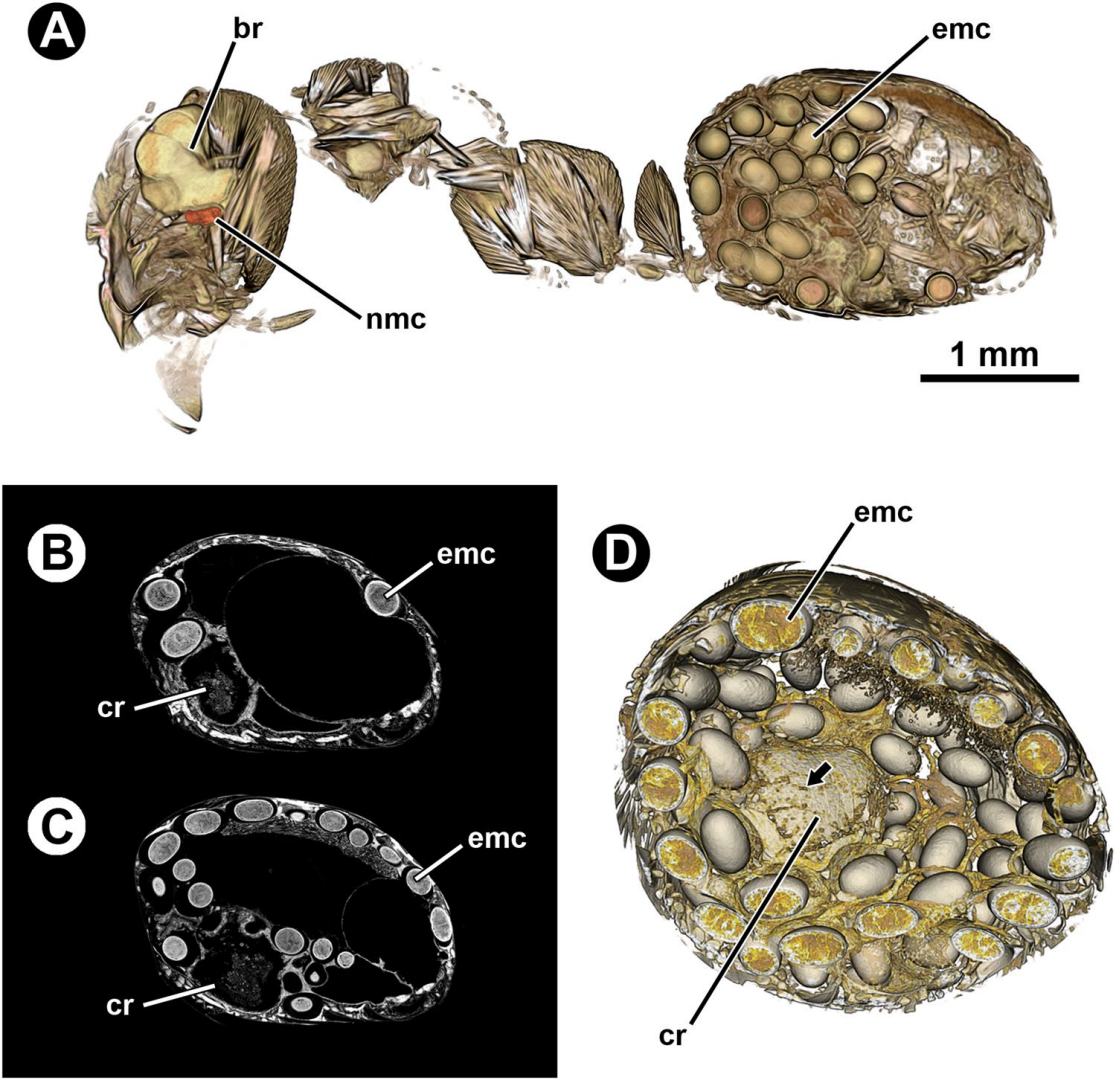
(**A**) False-coloured 3D volume rendering of an infected ant (*Formica aserva*) in sagittal section. (**B**) Micro-CT-based virtual sagittal section of an ant gaster harbouring 6 encysted metacercariae. (**C**) Micro-CT-based virtual sagittal section of an ant gaster harbouring 98 encysted metacercariae. (**D**) False-coloured 3D volume rendering of an infected ant gaster in sagittal section, showing scar-like marks (arrow) on the crop surface. Abbreviations: br, brain; cr, crop; emc, encysted metacercaria; nmc, non-encysted metacercaria.

Micro-CT revealed the internal anatomy of the ant host, as well as *D. dendriticum* metacercariae in the SOG and gaster (Fig. 2A). Metacercariae intensity per ant gaster (N = 5, corresponding to 4 scanned gasters plus one full ant body) varied between 6 and 98 (Fig. 2B,C). Encysted metacercariae randomly selected from the two ant gasters with the lowest and the highest parasite density showed similar volumes both including and excluding the cyst wall (Table 1). These encysted metacercariae were unevenly distributed in the interior of the gaster, embedded between the cuticle and the hypodermal muscles and between the walls of the different portions of the digestive tract (Fig. 2B–D). 3D volume renderings showed several scar-like marks on the crop wall (Fig. 2D), likely produced during the penetration of cercariae following their ingestion. Those scar-like marks were observed in all the scanned gasters of infected ants (N = 5) but not in the scanned gasters of uninfected ants (N = 2).

**Table 1:** Comparative volumes of brain versus gaster *Dicrocoelium dendriticum* metacercariae in the ant *Formica aserva*.

Parasite form	N	Volume range (mm^3^)	Average volume (mm^3^) ± SD
Non-encysted metacercaria in brain	5	0.0011–0.0017	0.0013 ± 0.0002
Encysted metacercaria from gaster (including cyst wall)	20	0.0021–0.0032	0.0027 ± 0.0003
Encysted metacercaria from gaster (excluding cyst wall)	20	0.0012–0.0024	0.0018 ± 0.0003
Encysted metacercaria from head (including cyst wall)	2	0.0026–0.003	0.0027 ± 0.0002
Encysted metacercaria from head (excluding cyst wall)	2	0.0011–0.0012	0.0011 ± 0.00006

**Figure 3:**
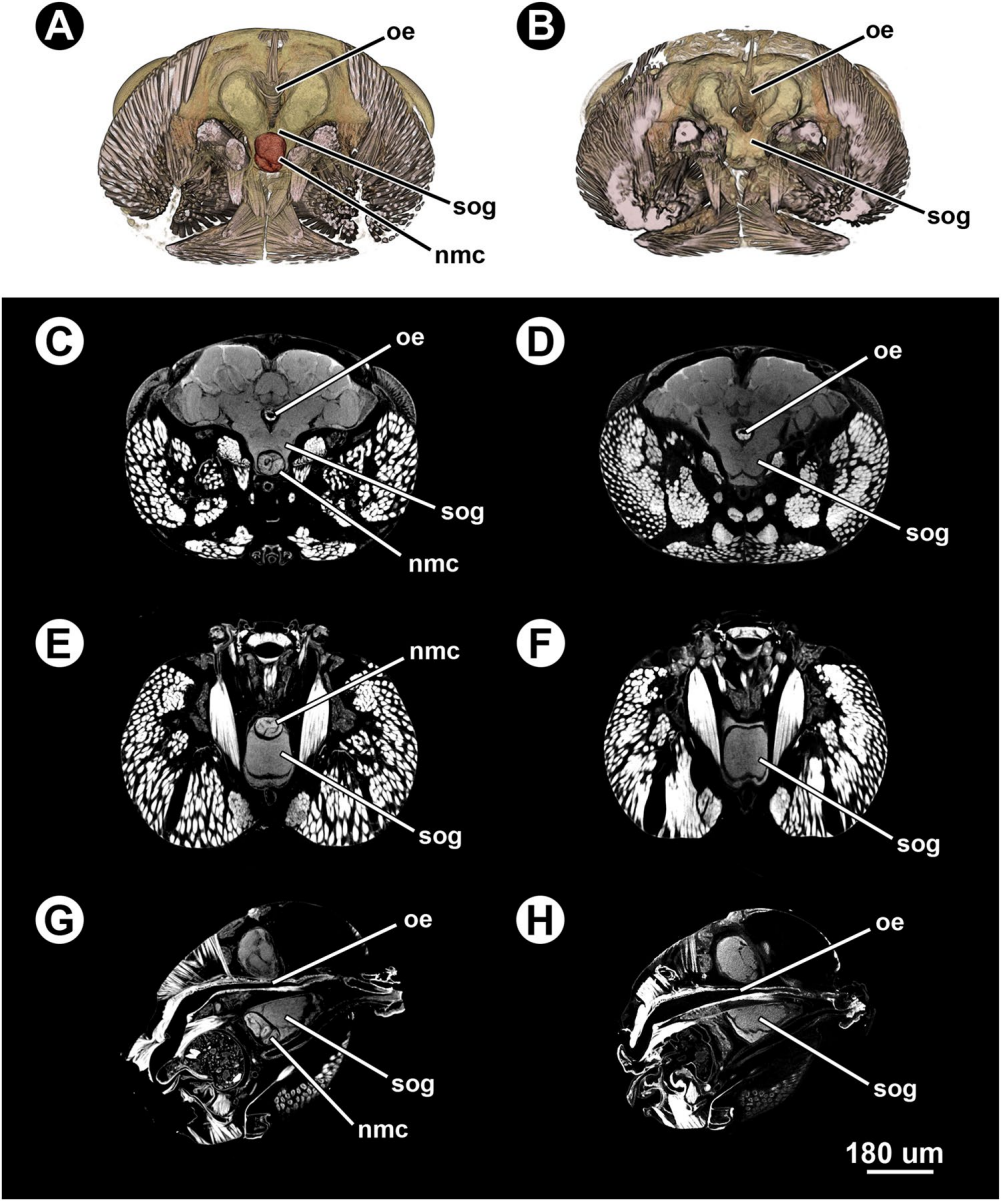
Micro-CT scans of the head of an infected (harbouring one non-encysted metacercaria) and an uninfected ant (*Formica aserva*). (**A**) False-coloured 3D volume rendering of the infected ant head in cross section. (**B**) False-coloured 3D volume rendering of the uninfected ant head in cross section. (**C**) Micro-CT-based virtual cross section of the infected ant head. (**D**) Micro-CT-based virtual cross section of the uninfected ant head. (**E**) Micro-CT-based virtual horizontal section of the infected ant head. (**F**) Micro-CT-based virtual horizontal section of the uninfected ant head. (**G**) Micro-CT-based virtual sagittal section of the infected ant head. (**H**) Micro-CT-based virtual sagittal section of the uninfected ant head. Abbreviations: nmc, non-encysted metacercaria; oe, oesophagus; sog, suboesophageal ganglion.

**Figure 4:**
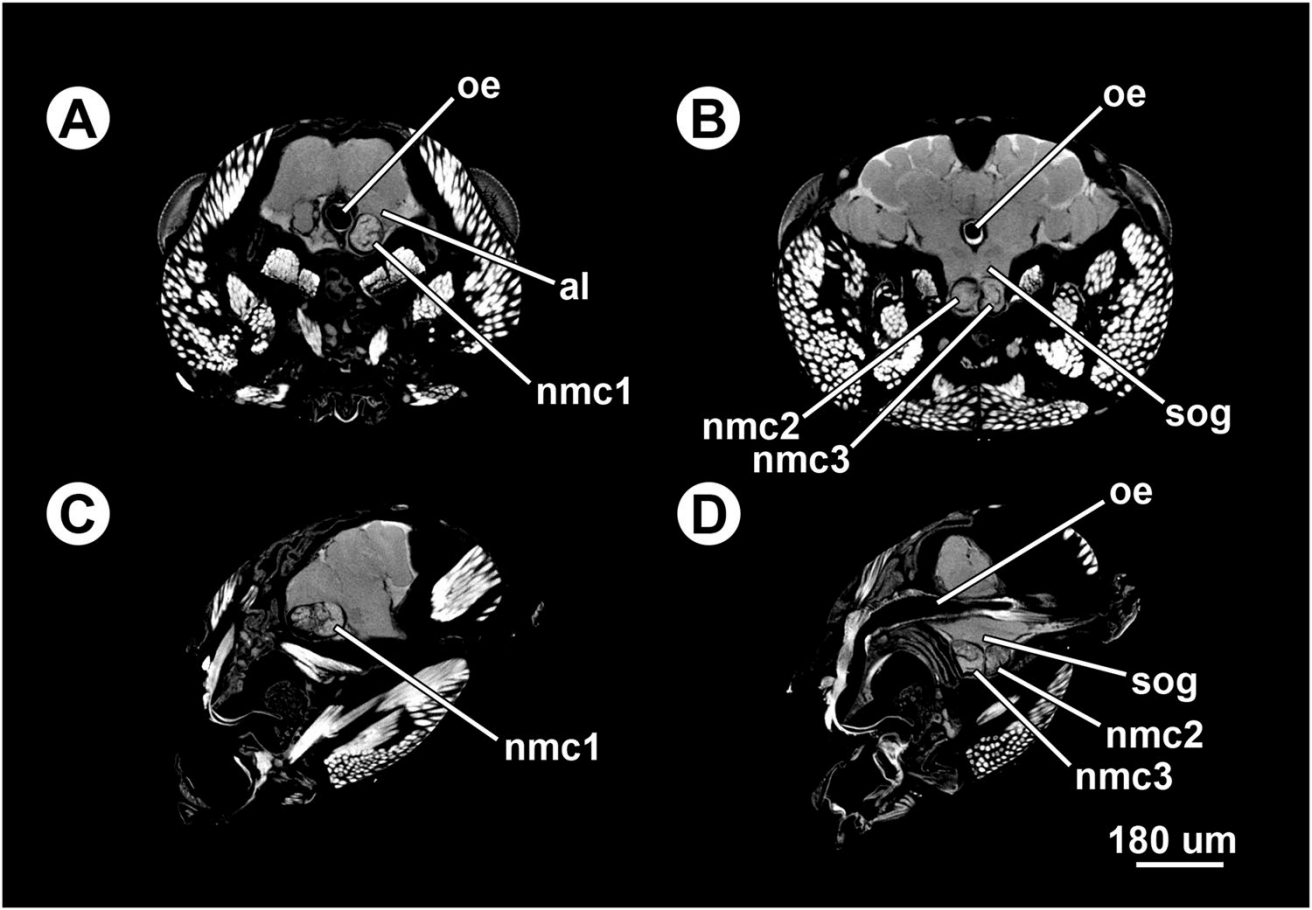
Micro-CT-based virtual sections of an infected ant head (*Formica aserva*) harbouring three non-encysted metacercariae (‘nmc1’, ‘nmc2’ and ‘nmc3’). (**A**) and (**B**), cross sections. (**C**) and (**D**), sagittal sections. Abbreviations: al, antennal lobe; nmc, non-encysted metacercaria; oe, oesophagus; sog, suboesophageal ganglion.

**Figure 5:**
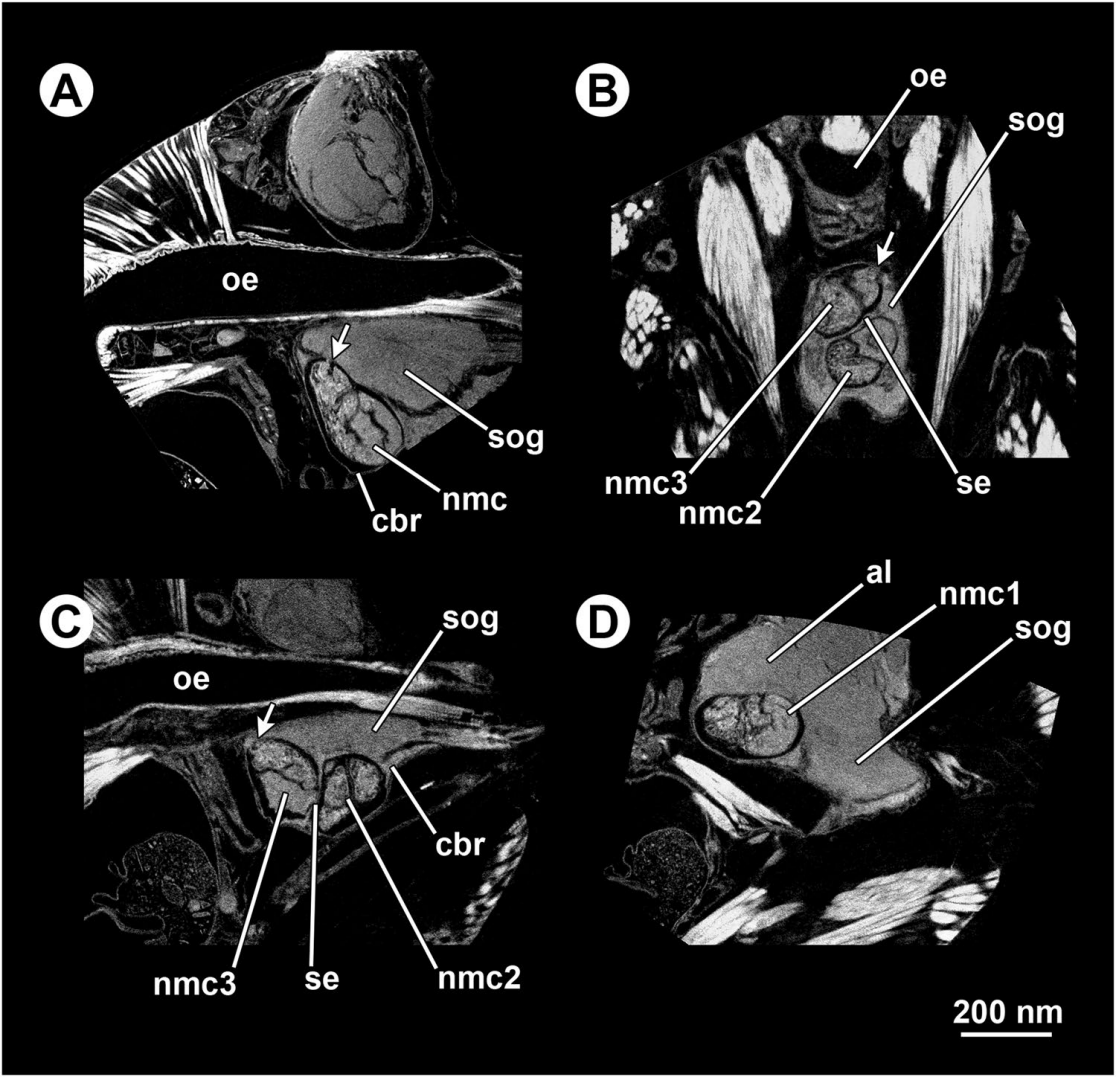
Micro-CT-based virtual sections of infected ant heads (*Formica aserva*), from scans zooming in on the non-encysted metacercariae. (**A**) Ant brain harbouring one non-encysted metacercariae in sagittal section; note the oral sucker of the parasite in contact with the host brain tissues (arrow). (**B**) Ant brain harbouring three non-encysted metacercariae in horizontal section, showing two (‘nmc2’ and ‘nmc3’) in the suboesophageal ganglion (SOG), separated by a thin septum of cell body rind; note the oral sucker of the most anterior one (‘nmc3’) in contact with the host brain tissues (arrow). (**C**) Ant brain harbouring three non-encysted metacercariae in sagittal section, showing two of them (‘nmc2’ and ‘nmc3’) in the SOG, separated by a thin septum of cell body rind; note the oral sucker of the most anterior one (‘nmc3’) in contact with the host brain tissues (arrow). (**D**) Ant brain harbouring three non-encysted metacercariae in sagittal section, showing one of them (‘nmc1’) between one of the antennal lobes and the SOG. Abbreviations: al, antennal lobe; cbr, cell body rind; nmc, non-encysted metacercaria; oe, oesophagus; se, septum of cell body rind; sog, suboesophageal ganglion.

Each of the ants that was collected attached to flowers was infected with at least one non-encysted metacercariae lodged within the anterior part of the SOG, between the surface of the neuropil and the surrounding cell body rind (Fig. 3A,C,E,G). No apparent alterations to SOG tissue were observed in comparison with uninfected brains (Fig. 3A–H). One of the infected ant heads was infected with three non-encysted metacercariae (Figs 4 and 5); one of them located between the SOG and one of the antennal lobes (Figs 4A,C and 5D; ‘nmc1’), and two lodged in the SOG (Figs 4B,D and 5B,C; ‘nmc2’ and ‘nmc3’). The two non-encysted metacercariae located in the SOG were close to each other but separated by a thin septum of cell body rind (Fig. 5B,C). The volumetric values of the non-encysted metacercariae were similar to, but slightly smaller, than those of the metacercariae from the gaster excluding the cyst wall (Table 1).

The ‘zoom’ scan on one non-encysted metacercaria from a randomly-selected infected ant head indicated that the oral sucker was in direct physical contact with the anterior part of the SOG (Fig. 5A). Another ‘zoom’ scan on the three non-encysted metacercariae lodged in a single ant brain showed that only the oral sucker of the one located in the most anterior part of the SOG was in direct physical contact with the brain tissue, unlike the oral sucker of the two remaining non-encysted metacercariae (Fig. 5B–D).

**Figure 6:**
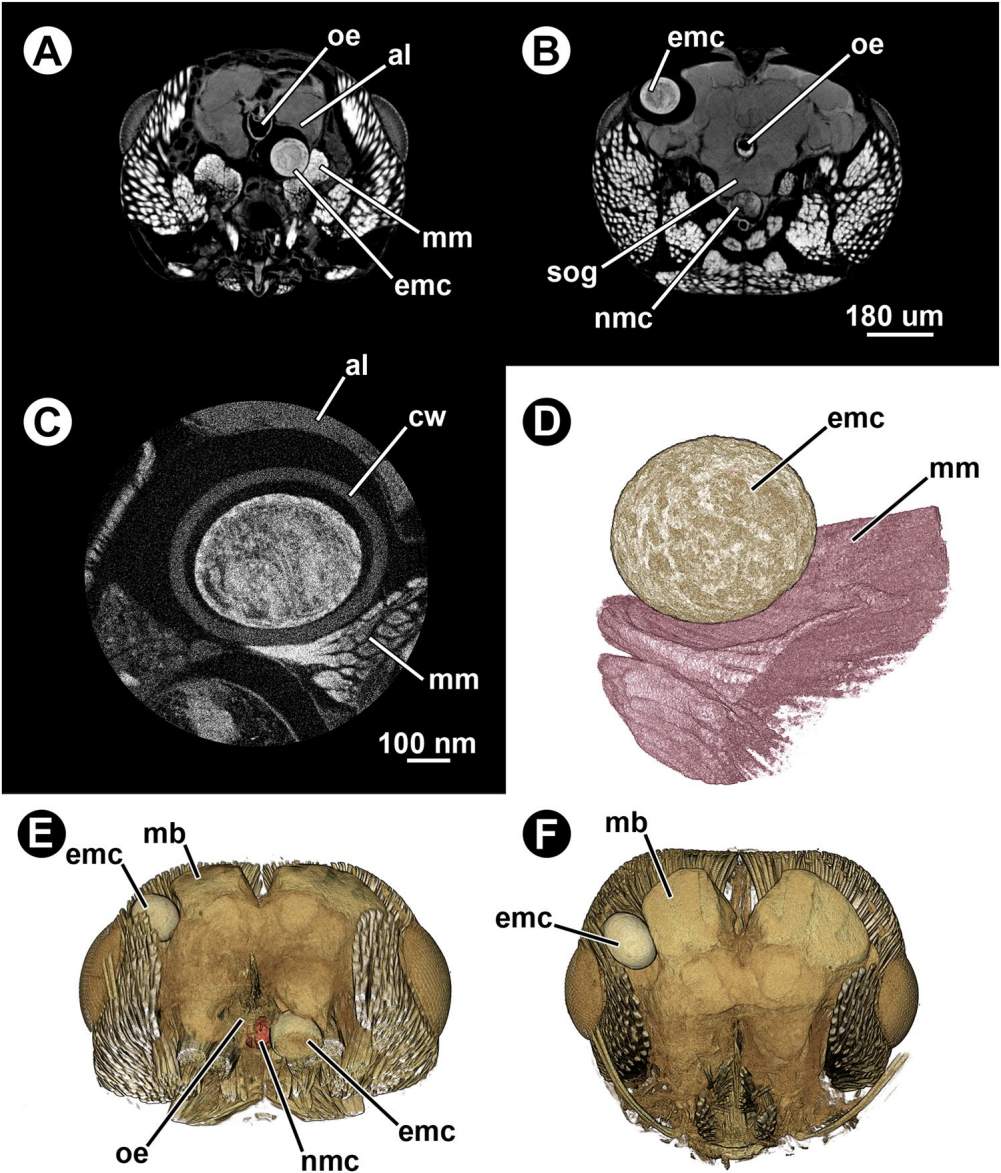
Micro-CT-based images of an infected ant head (*Formica aserva*) harbouring two encysted metacercariae. (**A**), (**B**) and (**C**), virtual cross sections. (**D**) False-coloured 3D volume rendering of one of the encysted metacercariae and the surrounding musculature. (**E**) False-coloured 3D volume rendering of the ant head harbouring two encysted metacercariae; cross section in dorsofrontal view. (**F**) False-coloured 3D volume rendering of the ant head harbouring two encysted metacercariae; horizontal view. Abbreviations: al, antennal lobe; cw, cyst wall; emc, encysted metacercaria; mb, mushroom bodies; mm, mandibular muscle; nmc, non-encysted metacercaria; oe, oesophagus; sog, suboesophageal ganglion.

One of the scanned infected ant heads showed, in addition to the typical non-encysted metacercariae lodged in the SOG, two encysted metacercariae whose structure resembled those observed in the gaster (Fig. 6A,B). The volumes of these encysted metacercariae were similar to those encysted within the gaster, whereas the volume values of the parasite alone (i.e. excluding the cyst wall) were similar to those from the smallest parasites (excluding the cyst wall) found in the gaster (Table 1). In that infected ant head, one encysted metacercaria was located between the cuticle and the calyxes of the mushroom bodies and the other between the SOG and one of the antennal lobes, visibly ‘denting’ the adjacent brain tissue and muscle fibres (Fig. 6A–F). Unlike the non-encysted metacercaria in the SOG, the two encysted metacercariae observed in the head were not surrounded by the cell body rind of the ant brain, therefore not in contact with the nervous tissue.

## Discussion

As a generalist parasite, *D. dendriticum* can be infective to a wide range of ant host species, although the intensity of such infection may vary among and within species^20,21^. The sizes and numbers of cysts in the scanned ants were comparable to those reported from other ant species by different authors (see Manga-González *et al*.^21^ for a review). Within species, the size of the ant might be one of the main factors influencing the number and volume of metacercarial cysts in the gaster, as it determines the amount of space available to the cysts. Nevertheless, Schuster^20^ observed a wide range in the number of cysts either in small-, medium- or large-sized ants, with significant differences only found between the smallest and largest individuals. The present observations are in line with those results as, even with a small sample size of only five scanned gasters of similar size, the range of the number of encysted metacercariae (6–98) was considerable. It is widely accepted that the behavioural changes shown by parasitised ants are independent of the intensity of the infection with encysted metacercariae^9,22^.

The results of CT scans of the heads of *F. aserva* found attached to flowers indicate that at least one *D. dendriticum* non-encysted metacercaria is consistently found within the ventral-anteriormost region of the SOG. Ants collected from the same nest, but not found attached to vegetation, were uninfected. Thus, the radical manipulation of ant behaviour that involves attachment to vegetation requires the presence of at least one non-encysted metacercaria in this region of the SOG. Site-selection and development of non-encysted metacercariae within the SOG can provide a direct pathway for the manipulation of locomotory activity and also the functioning of the mandibular muscles. When female jewel wasps inject venom into the heads of their cockroach hosts, they specifically target the SOG to alter their host’s drive to move^23^. Further, dendrites of the 10–12 motor neurons that stimulate the fast muscle fibres of the mandibular closure muscles in ants share the same neuropil in the ventral-anteriormost region of the SOG^24^ – the precise location where non-encysted *D. dendriticum* metacercariae are located. Thus, by targeting this region of the SOG, *D. dendriticum* has the potential to directly interfere with host locomotion and/or the action of the mandibles.

The SOG is a major part of the nervous system-wide network for neuromodulation in insects, evidenced by the many different neuromodulators expressed in neurons that reside in this region of the brain^25–27^. The effects of these neuromodulators – e.g. octopamine and FMRFamide-like peptide – are diverse and far reaching, from flight modulation, visual dishabituation and olfactory learning to mediation of the fight-or-flight response and the control of aggression in the case of octopamine^25–27^. FMRFamide-like peptide is implicated both as a neurotransmitter and a neuromodulator and affects the nervous system as well as skeletal muscle^25^. Thus, any influence exerted by metacercariae on the activity of neuromodulatory neurons in the SOG could have far reaching consequences for the behaviour of infected ants. Intriguingly, closely-related trematodes that utilize ants as second intermediate hosts, but encyst in brain regions other than the SOG, induce different and specific behavioural changes in their hosts which do not involve clinging to the vegetation, but which nonetheless enhance the chances of being ingested by the final host^5,28^.

It is unlikely that the localisation of a non-encysted metacercaria within the SOG is solely responsible for the complex sequence of behaviours demonstrated by infected ants. If this were so, that localisation would have to explain both attachment and detachment behaviours and it would have to explain the daily time periods when infected ants are apparently behaving normally. Instead, some form of active manipulation, perhaps through mechanical and/or neuromodulatory mechanisms, must be responsible for the manipulation of ant behaviour^9^. Our results show that the metacercaria that occurs in the SOG is not encased in the double-layered cyst wall that is characteristic of the fully encysted larvae located in the gaster. Instead, a thin layer of host tissue envelopes the entire larva except for an anterior region that is in direct physical contact with the ventral-most region of the SOG. This raises the possibility for direct chemical and/or mechanical mediation that leads to reversible tetany of the mandibular muscles at the host/parasite interface in the SOG. Further research will use micro-CT scanning to image the brains of infected ants collected before and after attachment to determine if the non-encysted metacercaria actively makes contact with the SOG to induce and maintain tetany at certain temperature thresholds.

One of the five ants that we processed for CT-scanning contained two non-encysted metacercariae in the SOG. The same ant also harboured a non-encysted metacercaria between the SOG and one of the antennal lobes. Instances of multiple infections in the brain of *F. aserva* are rare^6,7^. The results of studies involving large samples of other species of ants collected attached to vegetation further indicate that multiple infections in the brain are uncommon^21,29^. However, Romig *et al*.^5^ reported up to three non-encysted metacercariae in approximately 30% of infected ants collected from sites in Germany, with a maximum of two located within the SOG. Romig *et al*.^5^ and Schneider and Hohorst^4^ also reported rare instances of encysted metacercariae located within the head and thorax. Our study demonstrated for the first time a case of multiple infection in *F. aserva* and revealed that only the non-encysted metacercaria lodged in the anterior part of the SOG was in direct physical contact with the host nervous tissue. This observation supports the idea that attachment behaviour requires at least one non-encysted metacercaria to be in direct contact with the region of the SOG where the mandible closer muscle motor neurons are located^9,24^. Laboratory exposures of ants to infective *D. dendriticum* cercariae, which are not yet feasible, are required before we can understand whether alteration in ant behaviour is similar for ants that contain one or more metacerariae in the SOG. More fundamentally, laboratory exposures of ants, followed by serial CT-scanning would accurately define the migratory pathway of recently-ingested cercariae to the SOG. Lastly, laboratory exposures of metacercariae into potential definitive hosts are required to determine whether the rare metacercariae that encyst within the head are infective.

Although much has been written about the manipulation of ant behaviour by *D. dendriticum*, few studies had tackled a direct visualisation of the parasite *in situ* to define the precise location of the parasite in the ant brain and the interface between the parasite and the host tissues. The present study not only confirms some previous observations of *D. dendriticum* metacercariae within its secondary host^5^, but also provides new insights into this parasite-host interaction, for the first time visualising both a physical contact between the parasite and brain tissue and the presence of encysted metacercariae in the head. Moreover, the current scans also imaged for the first time a case of multiple brain infection by *D. dendriticum*, where only the metacercaria lodged in the most anterior part of the SOG was in direct physical contact with the ant brain tissue. Our results also highlight the potential of micro-CT in parasitological research, in contrast to the limitations of invasive and more time-consuming techniques like traditional histology. Micro-CT-based 3D virtual histology provides unparalleled opportunities to better understand form and function in morphological studies of parasites *in situ*, creating virtual models that can be sliced in any plane^17,18,30^. With the development of scanning systems capable of the submicron voxel size level^15^, a wide range of both animal and protistan parasites may be potentially imaged within their hosts, further understanding their life cycles and developmental stages. Moreover, using micro-CT scanning, host organs and tissues that undergo alterations after parasite infection might be tracked and analysed both quantitatively and qualitatively. Finally, an important aspect of micro-CT scanning in combination with 3D computer models is the potential use of large-scale interactive 3D printed models and impressive images for public outreach, communication and education^30^, engaging the public with parasitology and making them aware of the relevance of emerging or neglected parasitic diseases.
